# Spatio-temporal-spectral imaging of non-repeatable dissipative soliton dynamics

**DOI:** 10.1038/s41467-020-15900-x

**Published:** 2020-04-28

**Authors:** Joseph C. Jing, Xiaoming Wei, Lihong V. Wang

**Affiliations:** 1 Caltech Optical Imaging Laboratory, Andrew and Peggy Cherng Department of Medical Engineering, Department of Electrical Engineering, California Institute of Technology, 1200 East California Boulevard, Mail Code 138-78, Pasadena, CA 91125 USA; 2 Present Address: School of Physics and Optoelectronics; State Key Laboratory of Luminescent Materials and Devices; Guangdong Engineering Technology Research and Development Center of Special Optical Fiber Materials and Devices; Guangdong Provincial Key Laboratory of Fiber Laser Materials and Applied Techniques, South China University of Technology, 381 Wushan Road, Guangzhou, 510640 China

**Keywords:** Mode-locked lasers, Nonlinear optics, Solitons

## Abstract

Dissipative solitons (DSs) are multi-dimensionally localized waves that arise from complex dynamical balances in far-from-equilibrium nonlinear systems and widely exist in physics, chemistry and biology. Real-time observations of DS dynamics across many dimensions thus have a broad impact on unveiling various nonlinear complexities in different fields. However, these observations are challenging as DS transitions are stochastic, non-repeatable and often strongly coupled across spatio-temporal-spectral (STS) domains. Here we report multi-dimensional (space *xy* + discrete time *t* + wavelength *λ*) DS dynamics imaged by STS compressed ultrafast photography, enabling imaging at up to trillions of frames per second. Various transient and random phenomena of multimode DSs are revealed, highlighting the importance of real-time multi-dimensional observation without the need for event repetition in decomposing the complexities of DSs.

## Abstract (web summary)

Due to three-dimensional, stochastic and non-repeatable transitions of dissipative solitons, it is challenging to monitor their full dynamics. Here, the authors resolve the dynamics by wavelength and along two spatial dimensions with up to trillions of frames per second using compressed ultrafast photography.

## Introduction

Dissipative solitons (DSs) with universal particle-like properties are localized waves in various nonlinear systems that widely exist in physics, chemistry, and biology^1^. DSs are sustained through continuous energy exchange with the environment, wherein dynamic balances occur between dispersion (or diffraction) and nonlinearity, as well as gain and loss^2^. In general, the formation of DSs is multi-dimensional, including space and time, which leads to the complex nature of DSs. Within ultrafast lasers, DSs have proven to be an excellent and widely adopted platform to generate energetic single-mode (SM, one dimensional (1D)) mode-locked fs pulses^3^. Recently, three-dimensional (3D) solitons have also been discovered in multimode fibers (MMFs)^4,5^ and spatio-temporal mode-locking (STML) MMF lasers^6^, wherein many transverse and longitudinal modes were simultaneously locked together—resulting in complex structures that arise from spatio-temporal-spectral (STS) interactions^5–8^. The complex mechanisms within 3D solitons contradict with the intuitive understanding that strong modal dispersion in MMFs can lead to temporal walk-off among transverse modes. Apparently, there are still open questions on the nature of 3D solitons, and exploring their full scope requires real-time observation in all dimensions. In addition, in the presence of noise^9^, 3D solitons exhibit complicated instabilities and dynamics when detuned from their steady states. As a result, the observation on the dynamics of 3D solitons not only can provide key insights into open questions of various optical nonlinear phenomena but also enable tackling of challenging cross-disciplinary experiments on Bose–Einstein condensates, plasmas, polymers, fluids^10,11^, etc. Despite these exciting opportunities, observing ultrafast dynamics of 3D solitons, however, remains challenging^2,6^. Fully exploring time-varying optical 3D solitons furthermore requires an overall temporal resolution down to ps^12^, multiple degrees of freedom^4^, and a long recording length^13,14^, as transient DSs are typically unpredictable and non-repeatable.

Notably, real-time measurements of non-repeatable laser dynamics have recently been realized through single-shot technologies, such as temporal stretching^15,16^, temporal lensing^17–19^, and other variants that enable full-field characterization^18–20^. Intriguing nonlinear phenomena have also been discovered about optical rogue waves^21^, soliton molecules^2,14^ and their internal motion^16^, mode-locking dynamics^13^, and other stochastic processes^22,23^, to name a few. Yet, despite the importance of these findings, they are mainly confined to 1D phenomena and are unable to fully observe multi-dimensional DSs.

Inherently, a mode-locked laser usually delivers an ultrashort pulse train with an extremely low duty cycle (typically 10^−5^), as the mode-locked pulse (temporal width of 100s of fs) circulates within the laser cavity with a round-trip time on the order of 10s of ns. This scenario leads to a high degree of temporal sparsity, in which there is little (or even no) signal between the mode-locked pulses, providing a strong potential to apply compressed sensing techniques. We here present an ultrafast multi-dimensional imaging technology—STS compressed ultrafast photography (STS-CUP) operating at up to 2 trillion frames per second—to study the non-repeatable dynamics of MM DSs. In contrast to prior works, particularly CUST (compressed ultrafast spatio-temporal photography)^24^ and STRIPED FISH (spatially and temporally resolved intensity and phase evaluation device: full information from a single hologram)^25,26^, which focus on the observation of single-pulse behaviors, our system can not only capture the spectrally resolved mean modal profile (i.e., averaged over the full pulse duration) variation of a single 3D soliton but also enable the long-term observation on the round-trip modal dynamics as the 3D soliton circulates inside the MM laser cavity. These findings in this work will not only provide technical strategies to investigate various open questions on complex lightwave phenomena but also benefit various interdisciplinary studies.

## Results

### MM DS laser system

**Fig. 1:**
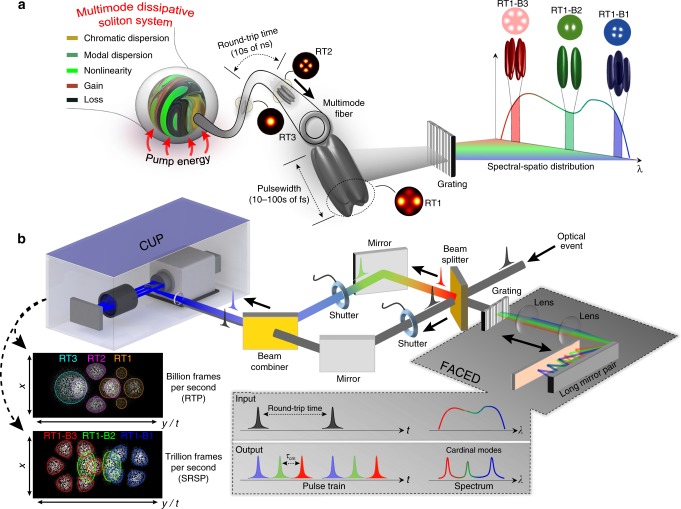
Spatio-temporal-spectral laser dynamics in a multimode DS system and schematic diagram of STS-CUP. **a** Multimode DS (dissipative soliton) system involving composite balances between multiple optical effects, where multiple transverse and longitudinal modes are simultaneously locked together to create ultrashort (fs) pulses. Inherited from the complexity of an optical dissipative system, a variety of spatio-temporal profiles can be generated over round trips, e.g., round trips 1–3 (RT1–3) illustrate different spatial cross-sections of 3D ultrashort laser bullets (typically 10–100 fs after dechirping). Owing to the nature of wavelength-dependent lightwave propagation in MMFs, a single pulse can exhibit different spatial profiles in different spectral bands (RT1-B1–3, note, the spatial profiles shown here are arbitrarily chosen for conceptual visualization, while much more complex cases could occur in reality, e.g., the one in Fig. 5), which can be resolved using optical gratings. **b** Schematic diagram of STS-CUP (spatio-temporal-spectral compressed ultrafast photograph). The fs optical event from the multimode DS system is split by a beam splitter into two branches that are respectively used for observations of round-trip pulses (RTPs) and spectrally resolved single pulse (SRSP). For the former case, the optical event is directly launched to the CUP unit (Supplementary Fig. 1). For the latter case, a single optical event is imparted with wavelength-dependent time delay through a free-space angular-chirp-enhanced delay (FACED) device. The optical spectrum of a single optical event is segmented into multiple spectral bands according to the cardinal modes of the FACED “cavity,” while each cardinal mode (spectral band) has a different time delay, typically 100s of ps (*τ*_cm_, right bottom inset). The spectrally resolved sub-pulses of a single optical event are then captured by the CUP unit. The STS-CUP system operates at speeds of billions or trillions of frames per second in RTP and SRSP modes, respectively.

While conventional DS fiber lasers work in the single transverse mode—delivering a Gaussian-like laser beam, a MM DS system (Fig. 1a) contains many transverse modes, each of which has a distinguishing spatial profile and propagation constant^6^. Particularly, the modal dispersion of MM waveguides (MMFs in this case) plays a contradictory role in the generation of MM DSs when working in the linear propagation regime. Consequently, in order to generate MM DSs, the lightwave must propagate in the nonlinear regime, where STS dynamics can be established through nonlinear effects of MMFs, e.g., self-phase modulation, cross-phase modulation, four-wave mixing, modulation instability, self-steepening, and Raman scattering. The complexity of such MM DS systems introduces tremendous challenges for probing their physical nature through direct experimental visualizations.

The MM DS system studied in this work is an STML fs MMF laser that has a similar scheme with ref. ^6^ (Supplementary Note 1). The laser has a ring cavity constructed from few-mode (FM) + MM fibers, where the gain fiber is cladding-pumped by a continuous-wave MM laser diode. The whole laser cavity has an all-normal dispersion, which prevents the generation of conventional solitons that usually propagate in the anomalous dispersion regime. Mode-locking is realized through nonlinear polarization rotation (NPR)^3^ implemented with a polarization-dependent transmission mechanism. A unidirectional operation via an isolator ensures that MM DSs can be self-generated by simply increasing the pump power.

### STS-CUP system

The STS-CUP system (Fig. 1b) has two channels that are utilized to capture the laser dynamics of round-trip pulses (RTPs) and spectrally resolved single pulses (SRSP), respectively. The input optical event is split by a 50:50 beam splitter. One output beam traveling through the RTP channel (gray color) is directly launched to the CUP unit (Supplementary Notes 2 and 3), which has a similar configuration with our prior works^27,28^, for capture of multiple RTPs within one acquisition. While operating in RTP mode, our system has a maximum imaging rate of 2 THz (500 fs between consecutive frames), set by the shear rate of the streak camera, which is too slow to characterize the dynamics within a single DS, which usually has a pulsewidth of 10–100 fs. In addition, broadband DSs (typically 10s of nm, Supplementary Fig. 1b) propagating in MMFs can generate speckle patterns with ultrafine features that vary with wavelengths down to a step size of 1 pm (i.e., ~300 MHz at 1.0 µm)^29^, resulting in an ultrahigh spatio-spectral diversity. Moreover, in DS cavities, thousands of longitudinal modes spaced at 10s of MHz over several THz, i.e., an optical frequency comb^30^, are simultaneously presented and phase-locked to form fs pulses. As a result, the STS propagation of DSs in MM systems can lead to dramatic dynamics, since the extremely high peak power of DSs can excite various nonlinear phenomena. Therefore, capturing the spectrally resolved (or even comb-line-resolved) spatio-temporal laser dynamics is essential for probing the physics of MM DSs. To this end, we extend our CUP system for STS imaging of a single fs pulse, i.e., the SRSP channel in Fig. 1b. Within the SRSP channel, the optical beam is sent into a free-space angular-chirp-enhanced delay (FACED)^31^ cavity, which segments the spectrum of the optical events and imparts a wavelength-dependent time delay (“Methods: FACED set-up”). The returned FACED signal is then relayed and co-propagates along the same optical path as the RTP channel toward the CUP unit after combining via another beam splitter. During imaging, complementary shutters in the RTP and SRSP paths control which channel is actively captured by the CUP unit.

### Validation of spatial mode visualization

**Fig. 2:**
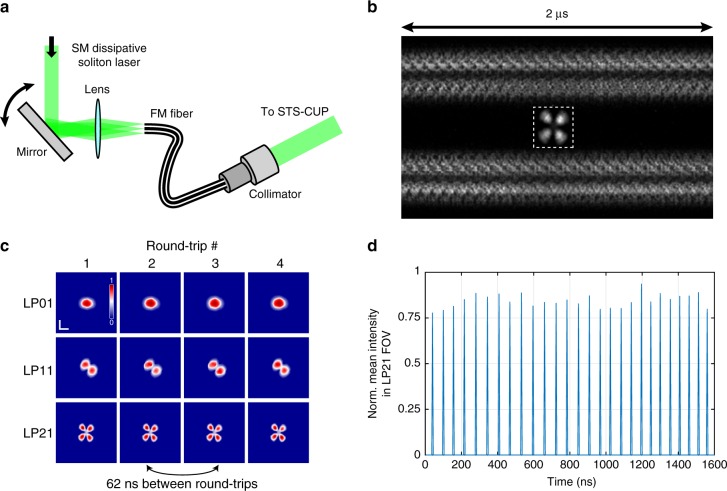
Generation and single-shot capture of stable linear polarization modes. **a** Schematic for generating stable LP (linear polarization) modes using single-mode DSs. The output from a SM (single mode) DS mode-locked fiber laser was coupled into a piece of FM (few mode) fiber (~1 m in length) using a scanning mirror and focusing lens. Changing the angle of the scanning mirror could excite different higher-order LP modes, which were then collimated and sent through the RTP channel for STS-CUP imaging. **b** Representative raw STS-CUP data acquired at a speed of 1 billion frames per second. Two horizontal bands of temporally sheared signal are the complementary views of a multimode DS train with LP21 spatial profile. The inset (dashed box) shows the corresponding temporally integrated image of the same multimode DS train captured by an external CCD camera. **c** Normalized round-trip DSs in LP01, LP11, and LP21 modes imaged by STS-CUP. Scale bar is 400 µm. **d** Normalized spatial mean intensity of the reconstructed STS-CUP images vs time plot of the reconstructed LP21 data set. The period of the reconstructed DS train is 62 ns, in excellent agreement with the round-trip time of the single-mode DS laser measured using a fast photodetector.

An SM DS fiber laser (1064 nm) was first constructed to validate our STS-CUP system’s ability to capture the spatial mode distribution of a series of round-trip DSs. The SM DS laser was built using all SM fibers (both passive and active ones) and had a cavity configuration similar to that of ref. ^3^. The fs DSs were generated through the NPR mode-locking mechanism. The generated pulse train was then coupled into a length of FM fiber (~1 m) through a scanning mirror and focusing lens (Fig. 2a). By adjusting the angle of incidence, we could selectively excite various spatial modes supported by the FM fiber (LP01, LP11, LP21, etc.). These generated spatial modes were then collimated and directed to the CUP unit through the RTP channel of our STS-CUP system for capture. As our CUP unit has an optimal sensitivity in visible wavelengths as determined by the photocathode within the streak tube, the center wavelength of the fs pulses was frequency-doubled to about 532 nm through second-harmonic generation (SHG) in a thin nonlinear crystal (BBO, ~20 μm thickness, not shown in Fig. 2a), which can maintain the spatial profile with a reasonable consistency (Supplementary Fig. 7). Please note that the directness of the real-time observation can be improved by eliminating the SHG and replacing the photocathode within the CUP unit with a near infrared one. Within the CUP unit (Supplementary Fig. 2), the pulse train was split into two complementary views and temporally sheared and integrated as described in Supplementary Note 3.

Figure 2b shows the raw data of a sequence of 27 complementarily spatially encoded and temporally sheared DSs having an LP21 spatial profile captured by our STS-CUP system through the RTP channel running at a frame rate of 1 billion frames per second. Please note that STS-CUP can operate at a flexible frame rate, from 1 million to 2 trillion frames per second, by simply changing the streaking speed of the camera. In general, a longer sequence of RTPs can be captured by STS-CUP by reducing the frame rate to compress more pulses within one capture; however, compressing too many pulses may necessitate demagnifying the spatial size of the optical pulses to maintain sparsity during processing (Supplementary Note 3). After reconstruction, the spatial profiles of 27 DSs were well resolved, with four spatial profiles of successive DSs shown in Fig. 2c (bottom row). It should also be noted that, using standard charge-coupled device (CCD) cameras, only ensemble summed images of spatial profiles over thousands of DSs can be obtained (inset of Fig. 2b), leaving the spatial mode dynamics over successive DSs indistinguishable. By changing the angle of incidence, the SM laser pulses were coupled into other spatial modes of the FM fiber and reconstructed, e.g., LP01 and LP11 as showcased in Fig. 2c. Given that the reconstructed data set is 3D in scale, we are also able to quantify the repetition rate of laser pulses, which was calculated to be 16 MHz (Fig. 2d) and found to be in excellent agreement with the value measured by a fast photodetector.

### MM DS dynamics

**Fig. 3:**
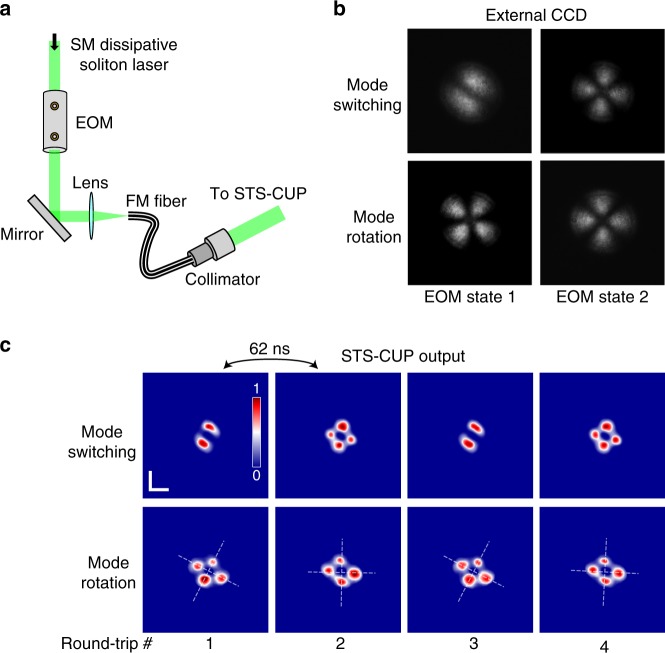
Generation and single-shot capture of dynamic linear polarization modes. **a** Schematic for generating dynamic LP (linear polarization) modes varying every round trip. The output from an SM (single mode) DS (dissipative soliton) mode-locked fiber laser was launched into a 30 MHz EOM (electro-optic modulator) acting as an ultrafast wave plate before being coupled into a length of FM (few-mode) fiber. The EOM operated in a binary fashion at a frequency equal to half of the repetition rate of the DS laser, i.e., 8 MHz in this case, such that every other DS generated a different spatial profile. **b** Generated spatial profiles as captured by an external CCD for comparison. In this case, the modulation frequency of the EOM was reduced to 1 Hz to match with the speed of the CCD, i.e., every 8 million DSs had the same spatial profile. Two different EOM states modulated at 1 Hz were demonstrated—mode switching and mode rotation; also see Supplementary Movies 1 and 2. **c** Round-trip DSs captured by STS-CUP for the same EOM states but modulated at 8 MHz. Scale bar is 400 µm.

We then investigated STS-CUP’s capacity to acquire a dynamic DS train with temporally differing spatial modes from pulse to pulse. To this end, we utilized a broadband electro-optic modulator (EOM) as a high-speed tunable wave plate to excite different spatial modes for successive round-trip DSs (Fig. 3a and “Methods: EOM mode control”). The output of the same SM DS fiber laser passed through the EOM before being coupled into a length of FM fiber. The EOM was driven with a square wave at a frequency equal to half the repetition rate of the DS laser (16 MHz), such that every sequential DS received a different phase rotation from the immediately preceding and following pulses. To excite specific spatial modes, the output of the EOM was coupled into the FM fiber again with an appropriate initial angle of incidence, yielding discrete spatial modes corresponding to the low/high states of the EOM. Generation of the two different dynamic schemes, mode switching and mode rotation, was first verified using a standard CCD by reducing the modulation frequency of the EOM driving signal to 1 Hz (Fig. 3b and Supplementary Movies 1 and 2). In the mode switching operation, the initial spatial profile of LP11 could be switched into an LP21 spatial profile. In the mode rotation scheme, on the other hand, we were able to demonstrate the rotation of an LP21 spatial profile by about 40°. Figure 3c shows STS-CUP reconstructions from the two respective operation schemes captured at an imaging speed of 1 billion frames per second. Both STS-CUP sequences show a clear distinguishment between sequential DSs in both mode switching and rotation operations with the spatial profiles in good agreement with the static profiles captured by the standard camera. Such high-speed active control and direct single-shot visualization of the spatial mode over successive DSs provide a potential technical solution for dramatically increasing the capacity of communication systems^32^.

Finally, we investigated the non-repeatable STS laser dynamics of MM DSs involved in an STML fs MMF laser using our STS-CUP system. The STML laser features a compact ring cavity design (Supplementary Fig. 1a). Briefly, the gain fiber, in the form of a length of FM double-cladding ytterbium-doped fiber (~4.5 m long), was cladding-pumped by an MMF laser diode (105 µm core size, 10 W maximum power) through a signal and pump combiner (SPC). The gain fiber has a core size of about 10 µm, which works in a weakly MM regime and supports the lowest four modes (LP01, LP11, LP21, and LP02). To enforce a strong MM operation, an MM graded-index (GRIN) fiber (62.5 µm core size, ~1 m long), which supports 100s of spatial modes, was fusion-spliced to the FM gain fiber with a core offset (by about 20 µm, right inset of Supplementary Fig. 1a). The use of GRIN fiber, in addition to supporting many modes, can largely reduce the modal dispersion to a comparable magnitude of the chromatic dispersion, which is essential for successful STML. After the GRIN fiber, the lightwave was launched into free space for optical manipulations, including spectral filtering, signal extraction, polarization control, and polarization-dependent transmission. The lightwave was subsequently coupled back to the cavity through a passive FM fiber with a core size matched with the FM gain fiber, which also served as a spatial filter that facilitates STML^6^. In addition to the spatial filtering, a bandpass filter was placed in the laser cavity for spectral filtering, in which way an arising pulse can meet the periodic boundary condition in both space and time domains to achieve stable STML.

**Fig. 4:**
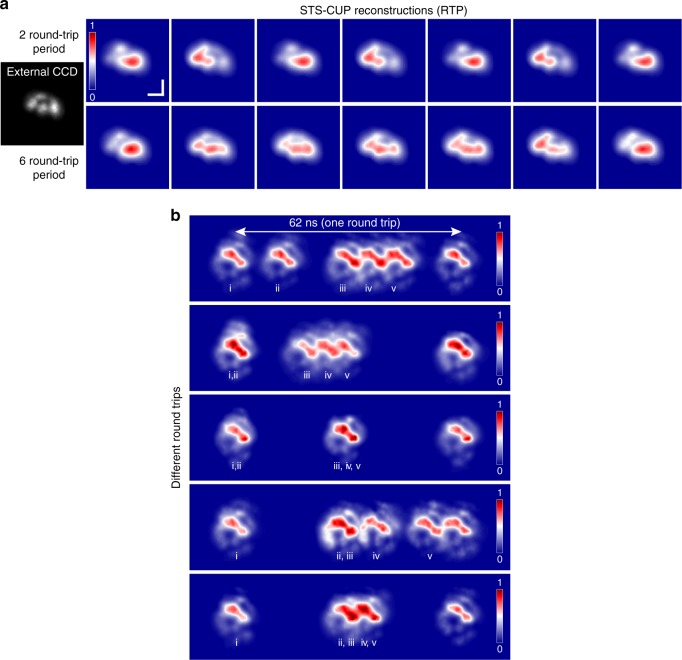
Stochastic laser dynamics of a spatio-temporal mode-locking laser. **a** Round-trip spatial profiles of successive 3D DSs with different evolution periods, i.e., two (top) and six (bottom) in this case, see Supplementary Movies 3 and 4. The left gray image shows the integrated spatial profile measured by an external CCD. Scale bar is 400 µm. **b** Temporal variation of multi-pulse solitons within the same round trip. Four addition solitons (ii–v) were generated as a result of increasing pump power. Attraction and repulsion of the solitons within the round-trip period was observed. Note that the laser cavity was not altered during the measurements.

The MM nature of STML DSs was first verified by using an external standard CCD, as shown in Fig. 4a (gray color image), where a complex spatial profile indicated that the mode-locked pulse was comprised of many transverse mode families. The time-averaged measurement of the spatial profile using the standard CCD camera exhibits relatively stable performance. The results captured by our STS-CUP, however, exhibit complex stochastic modal dynamics—consistent with the nature of rich spatio-temporal nonlinear interactions in MMF lasers. This observation largely implies that the MM laser was working in the partially STML regime, which is detuned from the optimal STML condition^6^. Not only did we find that the spatial profiles vary temporally but also that they could vary with a randomly changing interval or with a specific period. Figure 4a showcases two real-time modal evolutions. In the first case (top row), the round-trip DSs stably change sequentially between two spatial profiles. Meanwhile, in the second case, the round-trip DSs change across a time span of 2 µs with a periodicity that randomly varies between 2 and 6 round trips (Supplementary Movies 3 and 4). These spatio-temporal variations unpredictably, which occurred within the laser cavity with no external adjustments to the laser, highlight the necessity for ultrafast single-shot characterization technologies that require no event replication in DS dynamics studies over other pump–probe-based techniques^33^.

Careful experimental measurements have confirmed that STS-CUP is not sensitive to the state of polarization of the input laser beam (Supplementary Note 4 and Supplementary Figs. 4–6). Thus the physical origin of the modal evolution from pulse to pulse can be attributed to the dissipative nature of the MMF laser: the strong nonlinear interactions within ultrashort pulses in space, time and spectral domains serve as random perturbations that can introduce anisotropic propagation in MMFs, resulting in dynamic birefringence over the modal area. Such a stochastic intracavity birefringence can transform the state of polarization and thus the spatial profile of the circulating pulse over round trips^34^. Although the STML pulse was strongly localized in the time domain, evident by the ultrashort pulsewidth, fine confinement mechanisms for space and polarization were absent, given that the spatial filtering only relied on the FM fiber and there was no distributed polarizer along the non-polarization-maintaining MM laser cavity. This finding is also complementary to the round-trip spectral evolution reported for SM DS lasers^35^. Although it is not the focus of this work, further experimental studies are required for comprehensively understanding the physics behind such spatio-temporal laser dynamics in MM DS systems.

Complementary to the fundamental mode-locking in which a single soliton existed in the MMF cavity, we found that, by increasing the pump power or appropriately changing the state of polarization, the single soliton could be split into several, each with highly consistent intensities and spatial profiles (Fig. 4b). The coexistence of the multiple solitons in the same cavity exhibited complex interactions through attractive and repulsive forces. Figure 4b shows different random temporal patterns of multiple solitons within a round-trip period captured without any alterations of the laser cavity. The multiple solitons are observed to shift anywhere from up to five temporally separated pulses to as few as two distinguished pulses, demonstrating the dynamic nature of coexisting 3D solitons, which can be a fascinating platform for soliton molecule studies^36^. Please note that, while the observed round trips were captured sequentially over a period of 30 s, they are not consecutive round-trip events as the STS-CUP frame rate is much smaller than the repetition rate of our STML laser. The number of coexisting solitons were also found to vary when the parameters of the laser cavity were changed. For example, Supplementary Fig. 3 illustrates cases where 1, 2, and 3 solitons coexisted in the laser cavity when the pump power was gradually increased.

**Fig. 5:**
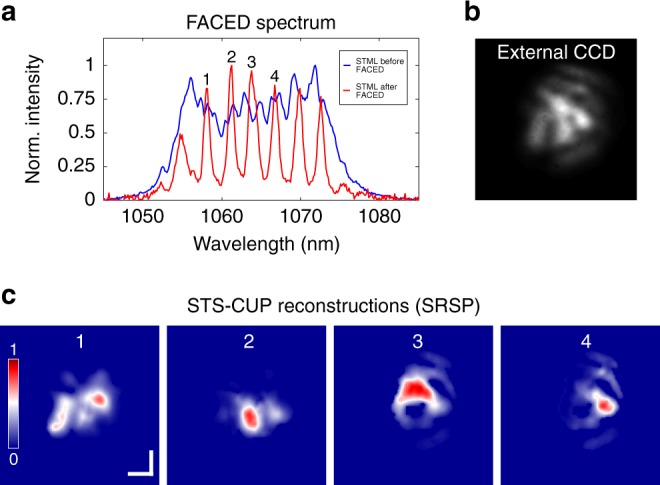
Spectrally resolved dynamics of a spatio-temporal mode-locking pulse. **a** Optical spectrum of 3D DSs discretely sampled by the FACED device of STS-CUP, where each peak/component corresponds to a cardinal mode of the FACED “cavity.” **b** Integrated spatial profile measured by an external CCD. **c** Spectrally resolved spatial profiles (SRSP) of a single 3D DS. Here only the modal compositions of four spectral components are given. Scale bar is 400 µm.

In addition to the DS dynamics over round trips, the multi-dimensional characteristics of a single DS can provide deeper insight into its fundamental physics. Here we imaged spectrally resolved modal compositions over the optical spectrum of a single DS (Fig. 5). The modal composition of each spectral component in the same DS, i.e., the sub-band discretely sampled by the FACED device of STS-CUP (red curve of Fig. 5a), was captured at a speed of 2 trillion frames per second, and the four spectral components were analyzed in Fig. 5c, where the integrated view captured by an external CCD is also given in Fig. 5b. The variation of the modal composition over the spectrum is evident. Please note that, if necessary, a much higher spectral resolution, potentially down to 10s of pm, can be obtained by upgrading the FACED device^31^. These results manifest that the spectral components of an STML pulse are MM and vary with wavelength since the involved modal compositions have time-varying phases or amplitudes across the spectrum. Further theoretical and experimental investigations can lead to a better understanding of these dynamics.

## Discussion

While STS-CUP provides attractive capabilities for studying complex STS DS dynamics, some constraints must be taken into careful consideration. The compressed sensing principle that STS-CUP leverages is well suited for capturing periodic and sparse optical events involved in STML MM lasers, in which the STML pulse with sub-ps pulsewidth fires in a period of 10s of ns, resulting in a high degree of temporal sparsity, i.e., a very low duty cycle on the order of 10^−5^. However, to gain a better understanding of the constraints in applying STS-CUP to 3D STML laser dynamics, one could conceptually visualize a potential experiment designed to capture the collision dynamics of multiple MM DSs. If the collision duration occurs over only a few round-trip cycles, then a high streak rate can be used. In this case, since the number of round-trip cycles and thus pulse events is relatively small, a high degree of sparsity is maintained, allowing for successful reconstruction of each individual DS’s features. If the collision duration instead occurs over many round-trip cycles, e.g., 10s to 100s of cycles, the streak rate of the system would need to be reduced in order to capture the increased number of round-trip cycles. The spatial size of the DSs on the streak camera could potentially be reduced to compensate for the increased information compressed within the CUP image. However, the pixel resolution for each spatial profile would be reduced, which could limit the ability to resolve finer features, especially in the case of more complex solitons. In addition, as the temporal resolution scales with the streak rate of the camera, operating with a reduced streak rate leads to a diminished ability to resolve inter-round-trip dynamics between the solitons, especially when the number of DSs increases. In this study, we could successfully reconstruct the dynamics of up to 60 consecutive round-trip events when the streak camera was slowed down to a streak rate of 500 MHz. In addition, our STS-CUP system has a maximum volumetric (*x, y, t*) refreshing rate of 50 Hz, which hinders our ability to observe continuous dynamic evolutions over longer time periods.

We expect that STS-CUP will enable the exploration of potential new nonlinear interactions through the direct characterization of multi-dimensional DSs. In particular, recent efforts on 3D nonlinear optics^37–39^ in MMFs have highlighted many opportunities across a wide set of interdisciplinary fields, while the understanding and exploitation of the underlying intricacies are still in their infancy. In addition, STS-CUP can also serve as a powerful tool for studies of random bit generation^40^, optical wave turbulence (either nonlinear propagation of coherent optical fields or linear propagation of incoherent optical fields)^41^, random laser^42^, and spatio-temporal optics in disordered media^43^, as well as high-capacity communication (via STS multiplexing and demultiplexing)^32^.

## Methods

### FACED set-up

In FACED, the optical event is first diffracted by a diffraction grating (Thorlabs GR25-0610, 600 grooves/mm, 1.0 µm blaze), after which the spectral components are encoded with angular dispersion. The diffracted signal (the first order) is then relayed through a 4*f* system to a pair of long mirrors (customized from Thorlabs, 750–1100 nm dielectric coating, 10 × 150 m size, >99.5% reflectivity). The long mirror pair has a minutely misaligned angle, on the order of mrad, which is adjusted by a high precision rotational stage (Thorlabs PR01). On the incident surface of the first long mirror, diffracted beamlets from the grating converge and bounce between the long mirrors according to their own incident angles. The angles of incidence of the beamlets will be gradually reduced until reaching normal incidence. Thus, instead of escaping from the far end of the long mirror pair, these beamlets matching with cardinal modes of the FACED “cavity” will eventually return to the origin exactly along their incoming paths. Since the cardinal beamlets have different light paths (zig-zag trajectories), the returned sub-pulses of cardinal beamlets have different time delays. The spectral resolution (or the number of cardinal modes) of FACED is governed by the ratio of the cone angle of converging beamlets at the entrance to the misaligned angle of the long mirror pair. The time delay between sub-pulses of cardinal beamlets, on the other hand, is determined by the separation of the long mirrors.

### EOM mode control

Output pulses from an SM DS mode-locked fiber laser were subsequently launched through a broadband EOM (Conoptics M350-160) and a piece of FM fiber (CorActive DCF-UN-20/125-080) to generate temporally varying spatial profiles. The EOM operated as an ultrafast variable wave plate and was driven by using a high-speed digital amplifier (Conoptics 25D, DC-30 MHz bandwidth). Both the DC bias and modulation amplitudes of the amplifier were set by monitoring the spatial modes between two EOM states using a standard CCD (FLIR System CMLN-1352M-CS). During dynamic STS-CUP imaging, the amplifier was seeded by an external function generator (Rigol DG1022) that generated a square wave with a duty cycle of 50% and a frequency equal to half that of the laser pulse repetition rate, i.e., 8 MHz in this case.

## Supplementary information

Supplementary Information

Description of Additional Supplementary Files

Supplementary Movie 1

Supplementary Movie 2

Supplementary Movie 3

Supplementary Movie 4

## Data Availability

All data used in this study are available from the corresponding author upon reasonable request.

## Code availability

All custom codes used in this study are available from the corresponding author upon reasonable request.
