# Supplementary material for: Spatio-temporal-spectral imaging of non-repeatable dissipative soliton dynamics
Source: Nat Commun. 2020 Apr 28;11:2059. doi: 10.1038/s41467-020-15900-x (PMC7189376; doi:10.1038/s41467-020-15900-x)
Supplement: Supplementary file 1 — Supplementary Information [file 41467_2020_15900_MOESM1_ESM.pdf]

Supplementary Information

Spatio-temporal-spectral imaging of non-repeatable dissipative soliton dynamics

Jing et al.

Supplementary Note 1 — Spatiotemporal mode-locked fs multimode fiber laser

Supplementary Figure 1a shows the configuration of the spatiotemporal mode-locked (STML) fs multimode fiber laser (MMFL) that is three-dimensionally mode-locked by using nonlinear polarization rotation (NPR) technology[1]. The laser cavity has a ring shape and a similar schematic with the prior work[2]. The few-mode (FM) gain medium is a piece of double-cladding ytterbium-doped fiber (Thorlabs YB1200-10/125DC, ~4.5 m long). The FM gain fiber has a core size of 10 μm, a core/cladding numerical aperture (NA) of 0.08/0.48, and a cladding-pump absorption of 1.7 dB/m at 920 nm. By using cladding pump scheme, the FM gain fiber can provide a power efficiency up to 84%. The MMFL is pumped through a signal & pump combiner (SPC) by a 920 nm multimode fiber (100/125 μm) laser diode that has a maximum power of 10 W. The pump port of the SPC has a lead multimode fiber the same as that of the pump laser diode, while both signal and common ports have lead fibers matching with the FM gain fiber in terms of fiber core (10 μm) and NA (0.08). The state of polarization (SOP) of the laser beam is adjusted by a half-wave plate (λ/2) and a quarter-wave plate (λ/4), while a polarization-dependent isolator (ISO) is employed to provide polarization-dependent transmission mechanism that is required for NPR mode-locking. Two fiber collimators (FCs) are utilized to launch the laser beam to free space and couple it back into the fiber cavity. A 50:50 beam splitter (BS) serves as the output coupler to extract half of the laser energy for characterizations. Both the lead fibers of the SPC and the FM gain fiber can support the propagation of modes LP01, LP11, LP21 and LP02. To excite higher-order modes, a piece of the multimode graded-index (GRIN) fiber (62.5 μm core size, ~1 m long) is fusion-spliced to the gain fiber with core offset of about 20 μm, see the right inset of Supplementary Figure 1a. The multimode GRIN fiber can support a mode number of >1000. A bandpass filter (F, Semrock LL01-1064-12.5, ~4 nm bandwidth) is placed in the laser cavity to facilitate the self-started mode-

locking. To match with the operating wavelength of STS-CUP, i.e., visible region, the output of the MMFL is frequency-doubled through second-harmonic generation (SHG) in a thin barium borate (BBO) crystal.

The MMFL can be turned into STML operation by increasing the pump power exceeding 4 W under an appropriate setting of the SOP. The output average power of the multimode mode-locked laser is >600 mW. Supplementary Figure 1b shows the optical spectrum centered at 1064 nm. As shown in Supplementary Figure 1c, the STML pulse train has an interval of about 62 ns, corresponding to a repetition rate of 16 MHz. The dechirped pulse duration is measured to be about 130 fs (Supplementary Figure 1d).

Supplementary Note 2 — Principle of CUP unit

Our CUP unit closely follows previously reported implementations with the inclusion of an additional temporal intensity mask[3-5]. In contrast with other high-speed imaging technologies[6-9], three views of the input optical event are captured by the STS-CUP system. One time-unsheared view is recorded by an external CCD camera while two time-sheared views that are spatially encoded with complementary binary patterns are captured using a picosecond streak camera. Combined, these three views provide a total of two distinct lossless projections of the optical event. The three views can be mathematically described as:

$$
\begin{aligned}
E^{(0)} &= TF_0 I(x, y, t) \\
E^{(1)} &= TSD_1 F_1 C_1 I(x, y, t) \qquad (1) \\
E^{(2)} &= TSD_2 F_2 C_2 I(x, y, t)
\end{aligned}
$$

where $E^{(0)}$, $E^{(1)}$, and $E^{(2)}$ represent the single and dual optical energy distributions captured by the external CCD and streak camera, respectively, T represents spatiotemporal integration, F represents spatial low-pass filtering, S represents temporal shearing, D represents image distortion within the encoding arm, and C represents the complementary spatial encoding provided by the DMD. Supplementary Equation (1) can be combined into the form:

$$E = OI, \tag{2}$$

where $E = [E^{(0)}, \alpha E^{(1)}, \alpha E^{(2)}]^{\mathrm{T}}$, $O = [TF_0, \alpha TSD_1F_1C_1, \alpha TSD_2F_2C_2]^{\mathrm{T}}$ and α is a normalization factor relating the optical power distributions of the external CCD camera and the streak camera. To obtain our CUP reconstruction from Supplementary Equation (2), we employ the two-step iterative shrinkage/thresholding (TwIST) algorithm[10] to solve the optimization problem:

$$\hat{I} = \arg \min_{I \in I_S \cap I_T} \left[\frac{1}{2} \|E - OI\|^2 + \beta \Phi(I) \right], \tag{3}$$

where the first term $\frac{1}{2}\|E - OI\|^2$ represents the measurement fidelity, $\Phi(I)$ is the regularization term, and β is a weight ratio between fidelity and sparsity. For CUP reconstructions of spatial modes, we employ three-dimensional total variation as the regularizer. We confine the set of possible solutions, I_S, for our reconstructions by employing a binary spatial mask created from the external CCD view. In addition, we further confine the set of possible solutions by employing an additional temporal mask into the reconstruction algorithm provided by a high-speed photodetector that monitors the output of the laser cavity.

Supplementary Note 3 — Experimental setup of CUP unit

Our CUP unit is based around our previously reported lossless-encoding detection scheme. Collimated output pulses from the fiber laser cavities are coupled to a beam splitter that directs a portion of the light to a standard external CCD (FLIR System CMLN-1352M-CS), capturing a time unsheared view of the input optical event, while the remaining light is relayed with a tube lens and a stereoscope objective (150 and 50 mm focal lengths, respectively) to the surface of a DMD (Texas Instruments DLP3000). The DMD, encoded with a pseudo-random pattern, deflects the light into two complementary views which are collected by the same stereoscope objective. The two beams are then directed through two tube lenses (150 mm focal length) and directed by a pair of mirrors and a right angle prism mirror to form complementary images on separate horizontal spatial areas of the streak camera (Hamamatsu C7700, CMOS Hamamatsu Flash4.0 V2, 2048×2048 pixels), which has its entrance slit fully opened. Compared with our previous CUP systems, the upgraded camera sensor features a higher quantum efficiency and increased FOV in both horizontal and vertical directions, the latter of which allows for capturing of twice as many round-trip DS events within one CUP acquisition. During streak imaging, these views are temporally sheared along the vertical direction to form the final compressed image containing the spatio-temporal-spectral information about the input optical events. Our STS-CUP can operate at a speed from 1 million to 2 trillion frames per second, while we set it to 1 billion frames per second for round-trip pulses capture and 2 trillion frames per second for spectrally-revolved single pulse capture. For STS-CUP reconstructions, the spatial FOV captured by the streak camera is set to be 1024 pixels by 848 pixels in the x and y dimensions, respectively, allowing for a reconstruction of 1200 temporal pixels. Operating the system at lower frame rates results in the capture of more round-trip pulses within a single STS-CUP image, however increasing compression within an image also increases the spatial overlap of the optical pulses and challenges our sparsity constraint,

which can cause difficulties during reconstruction. These issues can be alleviated by reducing the spatial pixel size of the system along the *y* dimension while also optically demagnifying the captured pulses to reduce the degree of spatial overlap of round-trip events during the temporal integration process. The STS-CUP data is temporally spread across the entire 2048 pixels in the vertical direction of the streak camera, thus the raw image is subdivided into separate images. Each sub-image is then reconstructed to acquire a 3D datacube which are then stitched together, thanks to the periodicity of the STML laser, into a final datacube size of 1024 × 848 × 2048.

Supplementary Note 4 — Sensitivity of STS-CUP unit to the polarization of the input laser beam

To examine if our STS-CUP system is sensitive to the SOP of the input laser beam, we performed experimental investigations using an experimental setup shown in Supplementary Figure 4. In brief, to generate a multimode laser beam with continuously rotating linear polarization, the multimode laser beam was first passed through a polarizing beam splitter (PBS, can also be replaced with a polarizer), after which the SOP of the multimode laser beam was reinforced to be linear. Then, a half-wave plate (λ/2) was employed to continuously rotate the orientation of the linear polarization. Finally, the modal profile was captured by the CUP unit without mask encoding. Supplementary Figure 5 shows the CUP measurements of the multimode laser beam with continuously rotating linear SOP. As can be clearly observed, the modal profile is highly consistent, yielding an average two-dimensional correlation of >99.7%, as shown in Supplementary Figure 6, which implies that the STS-CUP system is not sensitive to the SOP of the input laser beam. Thus, the stochastic modal dynamics shown in Fig. 4a should have resulted from the STML laser itself, rather than from the STS-CUP system.

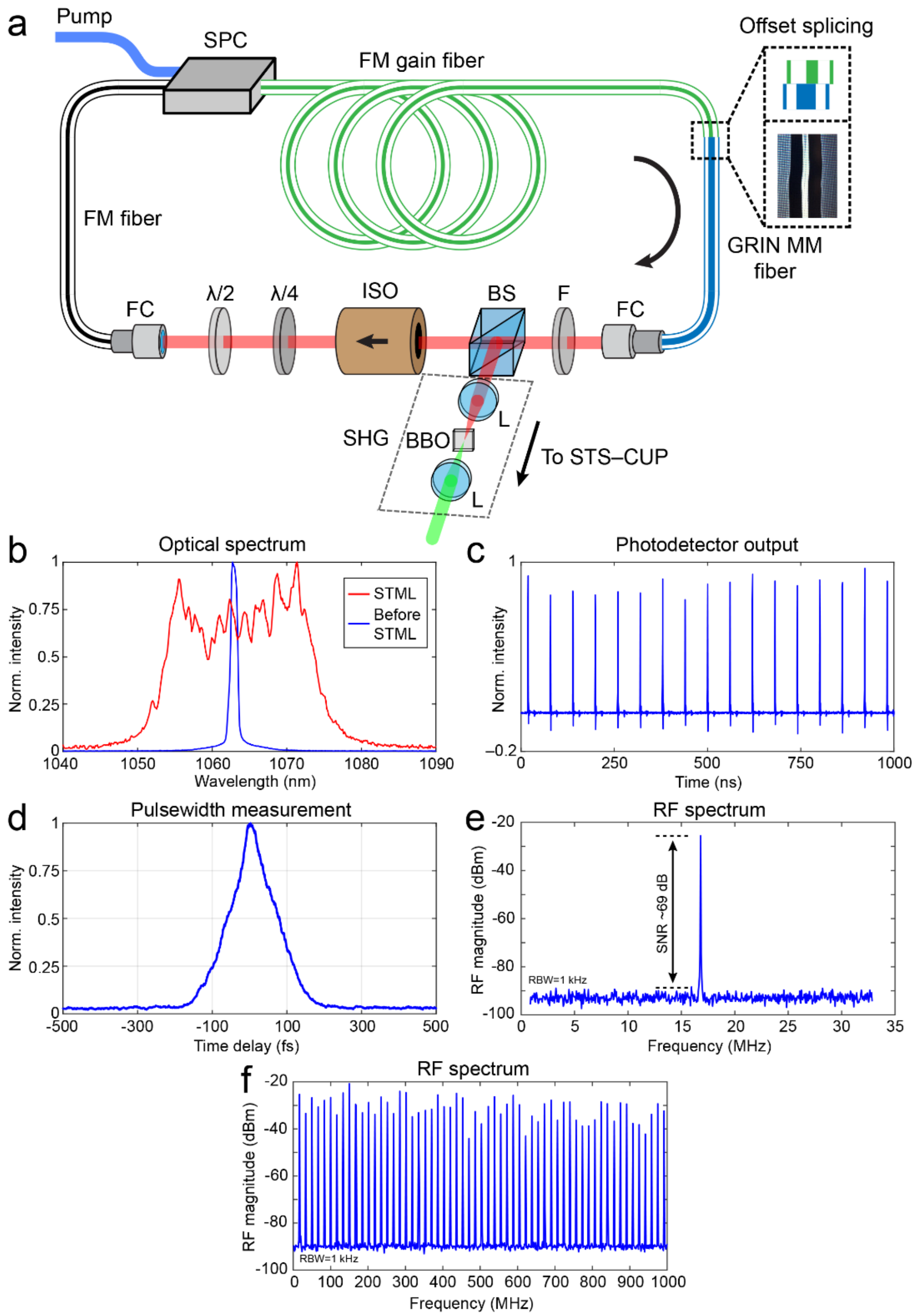

Supplementary Figure 1. Schematic and performance of the spatiotemporal mode-locking laser. a. Schematic of the spatiotemporal mode-locking (STML) fs multimode fiber (MMF) laser. BS: 50:50 beam splitter. F: bandpass filter. FC: fiber collimator. ISO: polarization-dependent isolator. L: lens. BBO: barium borate crystal. SHG: second-harmonic generation. SPC: signal & pump combiner. λ/2: half-wave plate. λ/4: quarter-wave plate. FM fiber: few-mode fiber. GRIN

MM fiber: graded-index multimode fiber. The right inset shows the offset fusion splicing for the excitation of higher-order modes. **b**. Optical spectrum of the STML pulses. **c**. Real-time pulse train of the STML laser. **d.** Pulsewidth measurement of the STML fs MMF laser after dechirping. It shows a full width at half maximum pulsewidth of ~130 fs. This measurement was performed with a conventional SHG-based autocorrelator, rather than a single-shot intensity autocorrelator. **e,f**. Radiofrequency (RF) spectral measurements with 32 MHz and 1 GHz frequency spans, respectively. A resolution bandwidth of 1 kHz was used for both measurements.

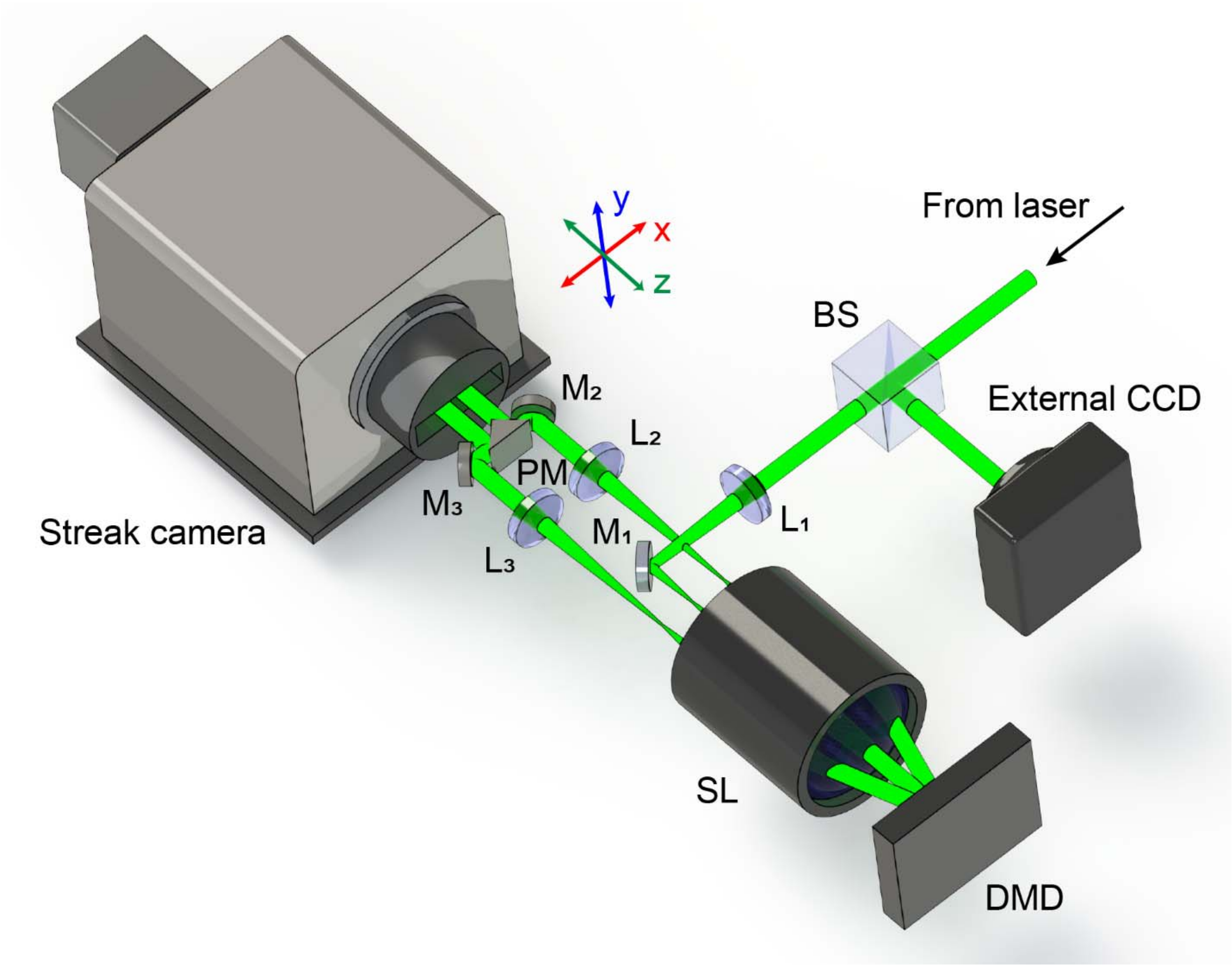

Supplementary Figure 2. Configuration of CUP unit. BS: beam splitter. DMD: digital micromirror device. L1–3: lenses, 150 mm focal length. M1–3: broadband mirrors. PM: prism mirror. SL: stereoscope objective lens, 50 mm focal length.

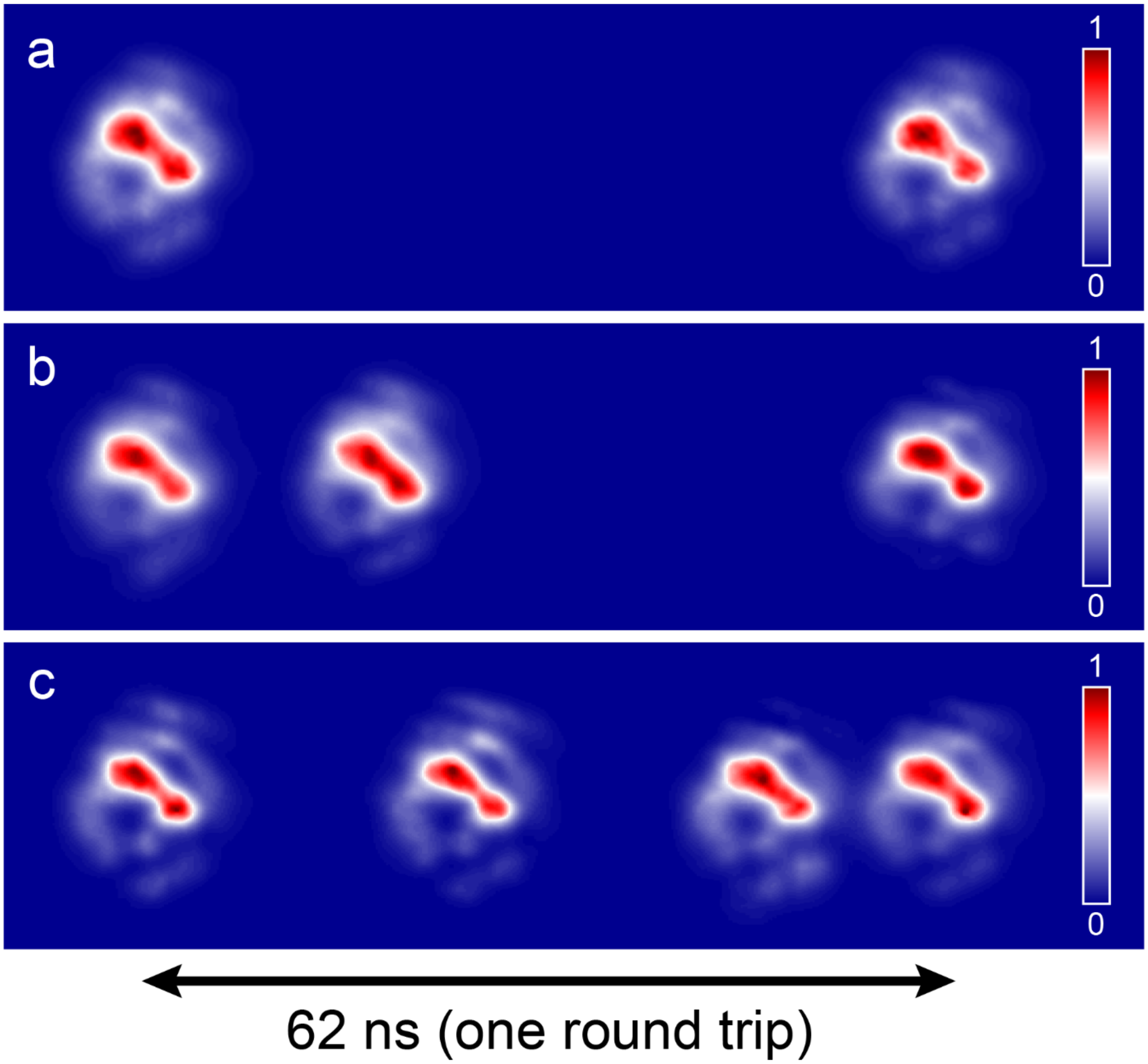

Supplementary Figure 3. Generation of multiple solitons within each cavity round trip with increasing pump laser power. The number of supported solitons increases from 1 to 3 when pump powers are increased from 4 W (**a**) to 4.5 W (**b**) to 5 W (**c**).

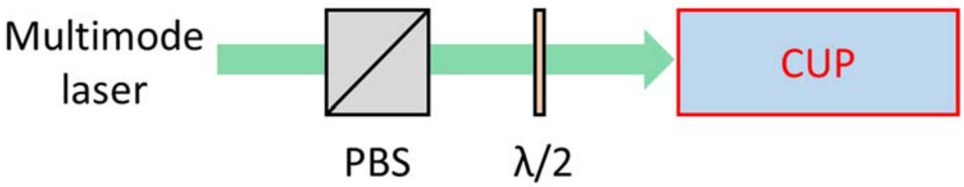

Supplementary Figure 4. Experimental setup for studying the sensitivity of STS-CUP to the state of polarization. PBS: polarizing beam splitter. λ/2: half-wave plate.

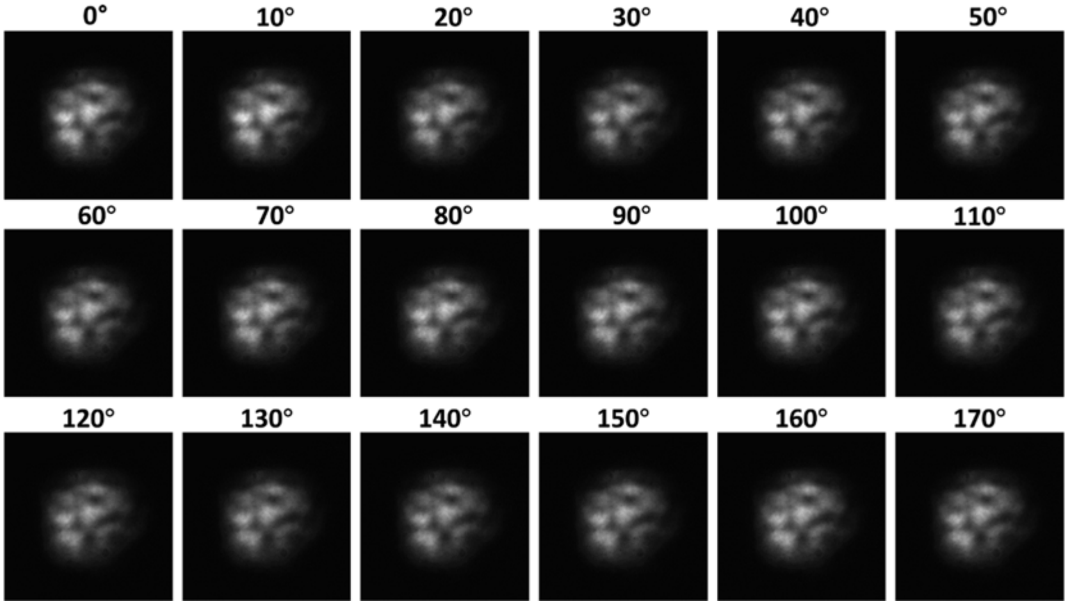

Supplementary Figure 5. STS-CUP imaging of polarization sensitivity. Modal profiles from a linearly polarized multimode laser captured by STS-CUP without mask encoding. A quarter-wave plate was used to rotate the linearly polarized light to characterize the polarization sensitivity within STS-CUP.

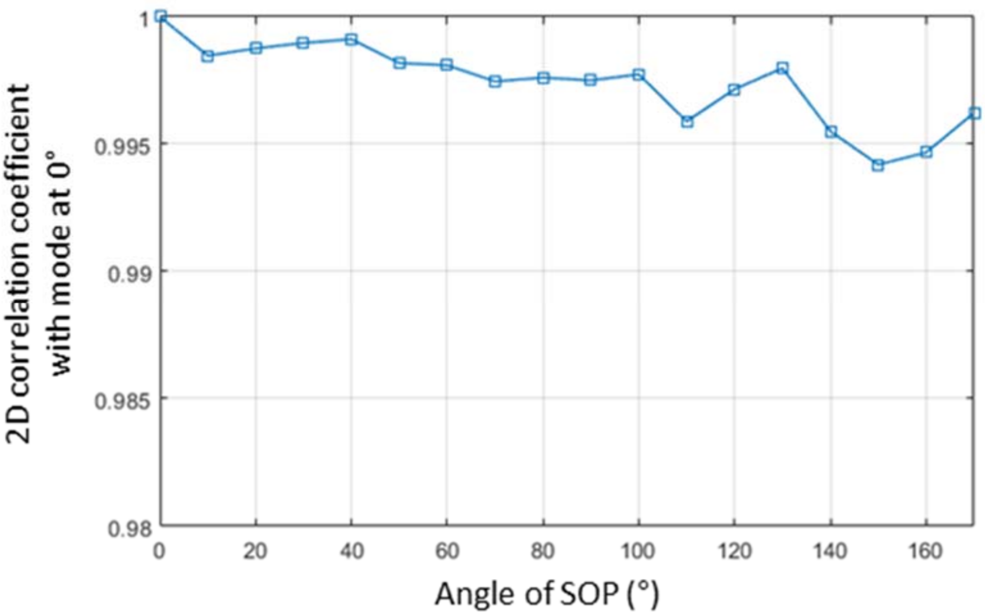

Supplementary Figure 6. Spatial correlation of modal profiles with different linear polarization states. 2D correlation coefficient calculated between the modal profiles from Supplementary Figure 5. The modal profiles remain highly consistent with an average 2D correlation of >99.7% which implies that the STS-CUP system is not sensitive to the SOP (state of polarization) of the input laser beam.

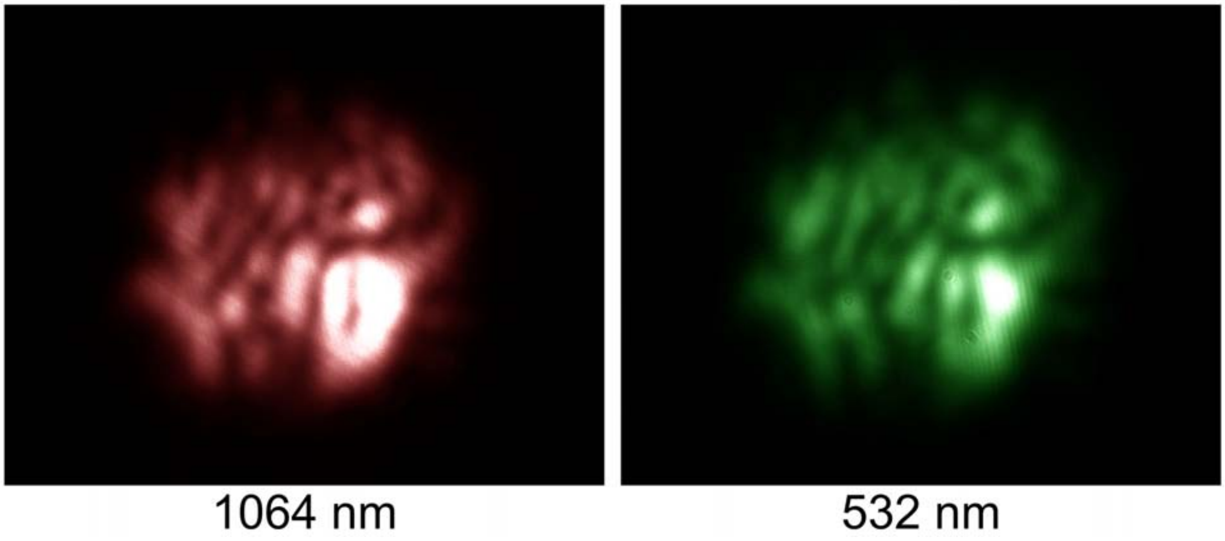

Supplementary Figure 7. Effects of second harmonic generation on spatial mode profile. Spatial mode profiles before (left, 1064 nm) and after (right, 532 nm) second harmonic generation (SHG) using a thin nonlinear crystal (BBO, ~20 μm thickness). Note here that, a much more complex modal profile was employed to better test the consistency of the spatial modes before and after the SHG.

Supplementary References

1. Chong A., Buckley J., Renninger W., Wise F. All-normal-dispersion femtosecond fiber laser. *Opt Express* **14**, 10095-10100 (2006).
2. Wright L. G., Christodoulides D. N., Wise F. W. Spatiotemporal mode-locking in multimode fiber lasers. *Science* **358**, 94-97 (2017).
3. Gao L., Liang J. Y., Li C. Y., Wang L. H. V. Single-shot compressed ultrafast photography at one hundred billion frames per second. *Nature* **516**, 74-77 (2014).
4. Liang J. Y., Ma C., Zhu L. R., Chen Y. J., Gao L., Wang L. H. V. Single-shot real-time video recording of a photonic Mach cone induced by a scattered light pulse. *Sci Adv* **3**, e1601814 (2017).
5. Zhu L. R., *et al.* Space- and intensity-constrained reconstruction for compressed ultrafast photography. *Optica* **3**, 694-697 (2016).
6. Wang X. F., Yan L. H., Si J. H., Matsuo S., Xu H. L., Hou X. High-frame-rate observation of single femtosecond laser pulse propagation in fused silica using an echelon and optical polarigraphy technique. *Appl Optics* **53**, 8395-8399 (2014).
7. Li Z. Y., Zgadzaj R., Wang X. M., Chang Y. Y., Downer M. C. Single-shot tomographic movies of evolving light-velocity objects. *Nat Commun* **5**, 3085 (2014).
8. Nakagawa K., *et al.* Sequentially timed all-optical mapping photography (STAMP). *Nat Photonics* **8**, 695-700 (2014).
9. Ehn A., Bood J., Li Z. M., Berrocal E., Alden M., Kristensson E. FRAME: femtosecond videography for atomic and molecular dynamics. *Light-Sci Appl* **6**, e17045 (2017).
10. Bioucas-Dias J. M., Figueiredo M. A. T. A new TwIST: Two-step iterative shrinkage/thresholding algorithms for image restoration. *Ieee T Image Process* **16**, 2992-3004 (2007).
