# Supplementary material for: Spatio-temporal-spectral imaging of non-repeatable dissipative soliton dynamics
Source: Nat Commun. 2020 Apr 28;11:2059. doi: 10.1038/s41467-020-15900-x (PMC7189376; doi:10.1038/s41467-020-15900-x)
Supplement: Supplementary file 2 — Description of Additional Supplementary Files [file 41467_2020_15900_MOESM2_ESM.pdf]

Description of Additional Supplementary Files

File Name: Supplementary Movie 1

Description: Generation of dynamic mode switching using a high-speed EOM. Output light from an SM dissipative soliton laser was launched through an EOM and coupled into a length of FM fiber before being collimated and imaged by a standard CCD camera. The EOM was operated in a binary fashion resulting in mode switching between LP11 and LP21 spatial modes. The EOM was driven at a rate of 1 Hz to allow for visualization of the mode changes by the standard CCD camera.

File Name: Supplementary Movie 2

Description: Generation of dynamic mode rotation using a high-speed EOM. Output light from an SM dissipative soliton laser was launched through an EOM and coupled into a length of FM fiber before being collimated and imaged by a standard CCD camera. The EOM was operated in a binary fashion resulting in the rotation of an LP21 spatial mode. The EOM was driven at a rate of 1 Hz to allow for visualization of the mode changes by the standard CCD camera.

File Name: Supplementary Movie 3

Description: Spatiotemporal visualization of round-trip pulses from our fully multimode mode-locked fs fiber laser. Spatial modes of the pulses from the cavity periodically alternate between every other round trip.

File Name: Supplementary Movie 4

Description: Spatiotemporal visualization of round-trip pulses from our fully multimode mode-locked fs fiber laser. Spatial modes of the pulses from the cavity periodically alternate with a randomly changing interval.
